# *Plinia trunciflora* Extract Administration Prevents HI-Induced Oxidative Stress, Inflammatory Response, Behavioral Impairments, and Tissue Damage in Rats

**DOI:** 10.3390/nu14020395

**Published:** 2022-01-17

**Authors:** Andrey Vinicios S. Carvalho, Rafael T. Ribeiro, Luz Elena Durán-Carabali, Ana Paula R. Martini, Eduarda Hoeper, Eduardo F. Sanches, Eduardo Luis Konrath, Carla Dalmaz, Moacir Wajner, Carlos Alexandre Netto

**Affiliations:** 1 Post-Graduation Program of Neuroscience, Instituto de Ciências Básicas da Saúde, Universidade Federal do Rio Grande do Sul, Porto Alegre 90046-900, Brazil; andreyscarvalho@outlook.com (A.V.S.C.); aprmartini@hotmail.com (A.P.R.M.); cdalmaz@ufrgs.br (C.D.); 2 Post-Graduation Program of Biochemistry, Instituto de Ciências Básicas da Saúde, Universidade Federal do Rio Grande do Sul, Porto Alegre 90460-060, Brazil; rafaelrated@hotmail.com (R.T.R.); ef.sanches@yahoo.com (E.F.S.); mwajner@ufrgs.br (M.W.); 3 Post-Graduation Program of Physiology, Instituto de Ciências Básicas da Saúde, Universidade Federal do Rio Grande do Sul, Porto Alegre 90050-170, Brazil; luzeled67@gmail.com; 4 Department of Biochemistry, Instituto de Ciências Básicas da Saúde, Universidade Federal do Rio Grande do Sul, Porto Alegre 90460-060, Brazil; eh-r@live.com; 5 Department of Pharmaceutical Sciences, Faculdade de Farmácia, Universidade Federal do Rio Grande do Sul, Porto Alegre 90610-000, Brazil; eduardo.konrath@ufrgs.br

**Keywords:** hypoxia–ischemia (HI), *Plinia trunciflora* extract (PTE), medicinal plants, antioxidants, spatial memory, neuroprotection

## Abstract

The disruption of redox homeostasis and neuroinflammation are key mechanisms in the pathogenesis of brain hypoxia–ischemia (HI); medicinal plants have been studied as a therapeutic strategy, generally associated with the prevention of oxidative stress and inflammatory response. This study evaluates the neuroprotective role of the *Plinia trunciflora* fruit extract (PTE) in neonatal rats submitted to experimental HI. The HI insult provoked a marked increase in the lipoperoxidation levels and glutathione peroxidase (GPx) activity, accompanied by a decrease in the brain concentration of glutathione (GSH). Interestingly, PTE was able to prevent most of the HI-induced pro-oxidant effects. It was also observed that HI increased the levels of interleukin-1β in the hippocampus, and that PTE-treatment prevented this effect. Furthermore, PTE was able to prevent neuronal loss and astrocyte reactivity induced by HI, as demonstrated by NeuN and GFAP staining, respectively. PTE also attenuated the anxiety-like behavior and prevented the spatial memory impairment caused by HI. Finally, PTE prevented neural tissue loss in the brain hemisphere, the hippocampus, cerebral cortex, and the striatum ipsilateral to the HI. Taken together our results provide good evidence that the PTE extract has the potential to be investigated as an adjunctive therapy in the treatment of brain insult caused by neonatal hypoxia–ischemia.

## 1. Introduction

Neonatal hypoxia–ischemia (HI) is one of the main causes of injuries affecting the Central Nervous System (CNS) in neonates [1]. HI is caused by gestational complications or events that occur after birth [1,2] and promotes unfavorable conditions for brain development, which contribute to the emergence of motor and cognitive impairments, emotional disorders, and various neurological diseases [3].

The pathophysiology of HI is caused by oxygen deprivation and the interruption of blood flow to the brain tissue [1,4]. Low concentrations of substrates such as glucose and oxygen lead to metabolic failure and the reduction of Adenosine Triphosphate (ATP) synthesis, which interferes with neuronal activity and promotes the activation of cell death pathways [5]. In addition, neuronal loss is also related to oxidative stress and subsequent neuroinflammation [1,6,7]. Mitochondrial metabolism is accelerated to compensate for the energy failure and high O_2_ consumption, which involves high respiratory chain activity and greater production of superoxide radicals by electron dispersion [8]. The increased production of oxygen reactive species overloads the antioxidant defense systems and leads to protein, nucleic acid, and lipid damage [8,9].

Neurological impairment is aggravated by the activation of the immune system and the recruitment of defense cells [10,11]. The acute inflammatory response in the CNS begins with the infiltration of peripheral leukocytes and the activation of microglia and astrocytes. Glial cells detect damage-associated molecular patterns (DAMPs) and become reactive, acting as secretors of pro-inflammatory cytokines [12]. Cytokines released by glial cells, including interleukins (IL-1, IL-6, IL-8, IL-10) and TNF-α, work as cellular signaling factors for inflammation; interleukin-1β (IL-1β) promotes the aggravation of the inflammatory response and consequently the mobilization of defense cells throughout the body to the CNS, thus causing an increase in neuron death [11].

Indeed, HI causes severe damage to the CNS, and this is reflected in brain maturation and function [13]. Despite the current knowledge on HI injury mechanisms, the main treatment used in newborns suffering from HI is therapeutic hypothermia; however, it does not promote complete recovery, even in moderate cases [14]. New neuroprotective strategies are needed to prevent the damage caused by the HI insult and allow for longer life expectancy and better quality of life for affected individuals.

Medicinal plants acting within the CNS and promoting the improvement or stability of neurological diseases have been extensively studied [15]. As for HI, experimental studies have investigated the influence of plant extracts or bioactive compounds that can prevent or delay the injury process [16]. Among the natural substances studied, polyphenolic compounds such as flavonoids have shown neuroprotective effects against HI [17] and in other models of experimental neurological disorders [18,19]. Most compounds derived from natural agents bear anti-apoptotic, anti-inflammatory, and antioxidant effects, such as the *Punica granatum* extract, *Huperzia quadrifariata* extract, grape seed extract, and others [20,21,22]. Furthermore, studies using plant-derived compounds, such as resveratrol, coumestrol, and cannabidiol, among others [23,24,25], demonstrate beneficial effects in the prevention or treatment of HI consequences.

*Plinia trunciflora* (O. Berg) Kausel belongs to the Myrtaceae family and is a species of the jaboticaba tree that is native to Brazil [26,27]. This fruit contains several phenolic compounds that have important antioxidant activity, which can mostly be attributed to flavonols and anthocyanins concentrated in the fruit’s skin [28,29,30,31]. Furthermore, the characterization of *P. trunciflora* through mass spectrometry has revealed the presence of phenolic compounds, such as cyanidin-3-O-glycoside (C3G) and delphinidin-3-O-glycoside (D3G), which can interact with the electron transport chain and reduce oxidative/nitrosative damage [32,33,34,35,36,37]. It was observed that the *P. trunciflora* extract, in an in vitro study, promoted the reduction of oxidative/nitrosative stress in human lung fibroblasts through the modulation of mitochondrial function [31].

The aim of the present study was to assess the possible neuroprotective effect of the *P. trunciflora* fruit extract against the impairments caused by neonatal hypoxia ischemia. Based on previous reports on its antioxidant effects and its varied polyphenolic components, we hypothesized that the bioactive properties of the *P. trunciflora* extract would limit the HI lesion process and its consequences. The effects of the *Plinia trunciflora* extract on cognition, brain structures lesion volume, and some parameters of redox homeostasis and neuroinflammation were investigated in Wistar rats undergoing neonatal HI.

## 2. Materials and Methods

All experiments in this study comply with the standards proposed by the International Guiding Principles for Biomedical Research Involving Animals. Procedures were performed in accordance with the Principles of Laboratory Animal Care (NIH publication 85-23, 1985), Federal Law No. 11.794/2008, Normative Resolution No. 37 of 15 February 2018, and Normative Resolution No. 30 of 2 February 2016 of CONCEA-Brazilian Practice Guidelines for the Care and Use of Animals for Scientific and Didactic Purposes-DBCA. The experimental protocols used in this study were approved by the Ethics Committee on the Use of Animals (CEUA) of the Universidade Federal do Rio Grande do Sul (UFRGS, protocol #38571).

### 2.1. Animals

Wistar rat pups were obtained from the Department of Biochemistry colony at the UFRGS. Litters consisted of 8 pups (4 male and 4 female pups), and the puppies were housed in standard conditions (22 ± 2 °C, 12 h light/dark cycle). The offspring were kept with the mother rat until weaning at the postnatal day 21 (PND21), and all procedures were performed by experimenters trained in animal handling. The induction of the HI model was performed on PND 7; the animals were anesthetized with inhaled isoflurane (4% for induction and 1.5% for maintenance) and underwent a surgical procedure for occlusion of the right common carotid artery. After occlusion, neonates were placed in a hypoxia chamber and exposed to a hypoxic atmosphere (8% oxygen and 92% nitrogen, with a flow of 5 L/min) for 60 min at 37 °C. At the end of hypoxia, they remained for 15 min under heating for recovery and were later returned to the box of origin with their mothers. Animals in the Sham group (surgical control) were anesthetized and submitted to an incision and had the carotid artery exposed, with no occlusion nor hypoxia performed.

### 2.2. Experimental Design

**Figure 1:**
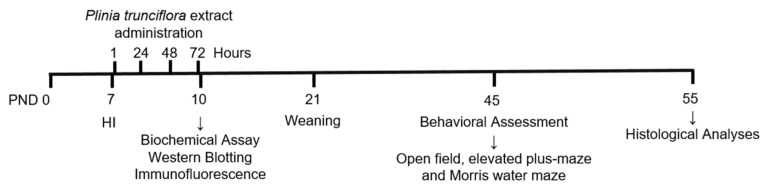
Experimental Design. From postnatal to adulthood in Wistar rats. PND: postnatal day; HI: hypoxia–ischemia.

The experimental design is presented in Figure 1. One hundred and nine animals on PND 7 were randomly allocated into four groups: Sham + saline (Sham + Sal), HI + saline (HI + Sal), Sham + *Plinia trunciflora* extract (Sham + PTE), and HI + *Plinia trunciflora* extract (HI + PTE). Intraperitonial (i.p.) injections of the extract or of saline were administered 1, 24, 48, and 72 h after HI. PTE was dissolved in sterile saline (0.9%) and administered at a concentration of 10 mg/kg in the Sham + PTE and HI + PTE groups; this dosage was chosen based on a previous study where the *Plinia trunciflora* extract showed antidepressant-like activity associated with antioxidant potential in vivo [38], and on the successful schedule of administration of another plant extract in the HI model [20]. The Sham + Sal and HI + Sal groups received i.p. saline injections according to their body weight. At 72 h after HI surgery (i.e., after the last injection of PTE), sixty-one animals were euthanized for biochemical (redox homeostasis parameters, *n* = 25, and western blot, *n* = 16) and immunofluorescence analysis (*n* = 20). Forty-eight animals were subjected to behavioral testing at PND 45 and were later euthanized for brain volumetric analysis. 

### 2.3. Preparation and Characterization of Plant Extract

The aqueous extract of *Plinia trunciflora* was obtained from the whole fruit. The fruit was crushed and lyophilized, after which it was extracted with distilled water acidified with HCl (pH 1.5) in an ultrasonic bath for 1 h. The solution was cooled to 4 °C for 12 h. After this period, the solution was filtered and lyophilized [39]. The lyophilized extract was kept in an amber bottle wrapped in aluminum foil in order to prevent the passage of light.

**Figure 2:**
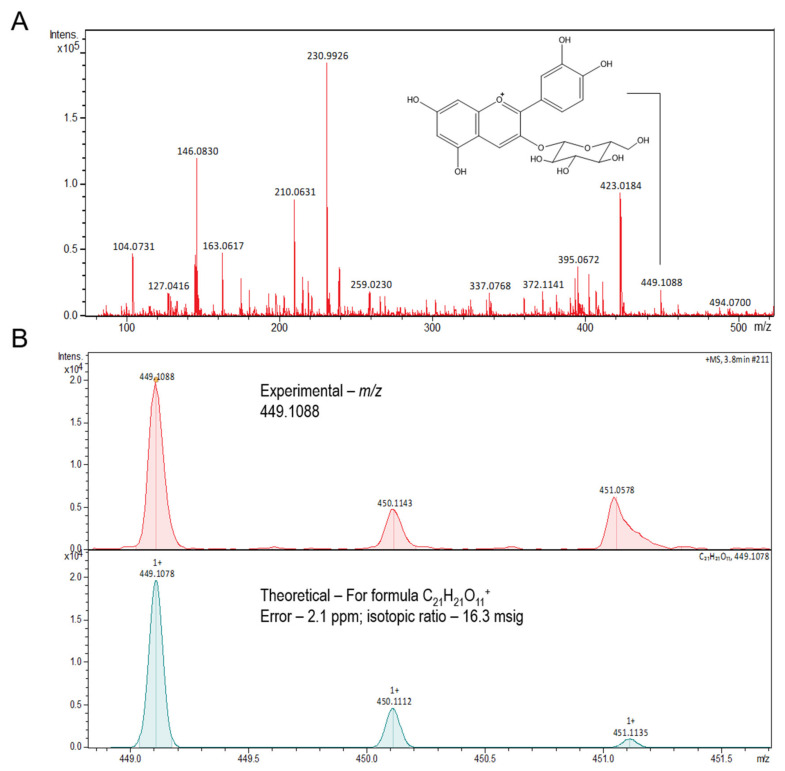
Full mass spectrum of the extract of *Plinia trunciflora* showing the compound cyanidin-3-O-glucoside (*m*/*z* 449.1088) (**A**). The tandem mass spectrometry (MS–MS) of cyanidin-3-O-glucoside (*m*/*z* 449.1088) from the extract of *Plinia trunciflora* (**B**).

The total polyphenolic content was determined by using the classic Folin–Ciocalteu colorimetric method, with some adaptations to the microplate assay [40,41]. The phenolic contents were expressed as mg gallic acid equivalents per 100 g dry weight (mg GAE/100 g dw). The assessment of the monomeric anthocyanin pigment was carried out using the pH differential method, with some minor modifications adapted to 96-well microplates [42]. Results are expressed as mg cyanidin 3-glucoside (MW = 449.2 g/mol, ɛ = 26,900 L/mol) equivalents per 100 g dry weight (mg cyn 3-glu/100 g dw), as shown in Figure 2.

As for the chemical analysis, the *P. trunciflora* extract was dissolved in a solution of 70% (*v*/*v*) chromatographic-grade ethanol (Tedia, Fairfield, OH, USA), 30% (*v*/*v*) deionized water, and 0.1% formic acid and infused directly into the electrospray ionization (ESI) source at a flow rate of 10 µL/min. ESI(+)-MS and tandem ESI(+)-MS/MS analyses were conducted on a hybrid high-resolution and high-accuracy Orbitrap mass spectrometer (Thermo Fisher Scientific Co, Waltham, USA), as previously described [31]. Diagnostic ions in the *P. trunciflora* extract were identified by the comparison of their ESI(+)-MS/MS dissociation patterns with compounds described in the literature. No important ions were observed below *m*/*z* 100 or above *m*/*z* 500, therefore ESI(+)-MS data are shown in the *m*/*z* 100–500 range. PTE peel and pulp are composed of Hexadecanoic acid, Kaempferol, Octadecanoic acid, and mainly Cyanidin-3-O-glucoside [31].

### 2.4. Redox Homeostasis Parameters

For the analysis of redox homeostasis, PND 10 animals were decapitated, and the hippocampus, cerebral cortex, and striatum ipsilateral to the carotid occlusion were dissected out and homogenized in 10 volumes (1:10, *w*/*v*) of 20 mM sodium phosphate buffer, pH 7.4, containing 140 mM KCl. Homogenates were centrifuged at 750× *g* for 10 min at 4 °C, and the supernatants were utilized for the determination of the parameters.

#### 2.4.1. Malondialdehyde (MDA) Levels

Lipid peroxidation was estimated by measuring the concentrations of MDA according to the method described by Yagi [43], with some modifications [44]. A total of 5–6 animals per group were used. Brain tissue supernatant (approximately 0.3 mg of protein) was incubated at 100 °C for 1 h in the presence of 10% trichloroacetic acid and 0.67% thiobarbituric acid in 7.1% sodium sulfate, and then cooled in water to room temperature. The resulting pink color was extracted with 400 µL butanol. The organic phase fluorescence was measured at an excitation wavelength of 515 nm and an emission of 553 nm. Concomitantly, a calibration curve was made using 1,1,3,3-tetramethoxypropane, in which all points were treated in the same way as the samples. Results were expressed as nmol TBA-RS/mg protein.

#### 2.4.2. Reduced Glutathione (GSH) Concentrations

The GSH concentrations were determined according to Browne and Armstrong (1998) [45], with some modifications. A total of 4–7 animals per group were used. Brain tissue supernatants (approximately 45 μg of protein) were deproteinized by adding 2% metaphosphoric acid and were centrifuged at 7000× *g* for 10 min. The supernatant was added to *o*-phthaldialdehyde (1 mg/mL in methanol) and incubated in a dark room for 15 min at room temperature. The resulting fluorescence was measured using excitation and emission wavelengths of 350 and 420 nm, respectively. A calibration curve was prepared using a GSH standard solution, and the results were expressed as nmol GSH/mg protein.

#### 2.4.3. Antioxidant Enzymes Activities

For the determination of the antioxidant enzyme activities, the samples were prepared in the same manner as that for the evaluation of other redox homeostasis parameters. A total of 4–7 animals per group were used. Brain supernatants (1.0–4.0 μg of protein) were utilized in these assays. The method based on the pyrogallol autoxidation by the superoxide anion was used to measure spectrophotometrically superoxide dismutase (SOD) activity at 420 nm [46]. The activity of glutathione peroxidase (GPx) was determined spectrophotometrically by monitoring the decrease in NADPH absorbance at 340 nm, using NADPH, GSH, and tertbutyl hydroperoxide as substrates [47]. The absorbance changes for all enzymes were recorded on a SpectraMax M5 plate reader (Molecular Devices, Sunnyvale, CA, USA). The specific activities were expressed as U/mg protein. These enzyme activities were calculated and expressed as units per mg of protein.

### 2.5. Western Blotting

Brains were quickly dissected out and the right hippocampus (ipsilateral to the carotid occlusion), was collected and stored at −80 °C. The tissue was homogenized and the protein concentration was determined. For this experiment, an equivalent protein concentration between the samples (40 μg of total protein) was used for running with NuPAGE^®^ 4–12% Bis-Tris Gels. Soon after electrophoresis, the proteins were transferred to nitrocellulose membranes, which were blocked for 2 h and incubated overnight, at a temperature of 4 °C, with the primary antibody. Anti-Interleukin-1β (1:1000, Abcam, #Ab9722), Anti-Interleukin-10 (1:1000, Thermo Fisher Scientific Co., Waltham, MA, USA, #ARC9102), and Anti-α-Tubulin (1:2000, Sigma-Aldrich Co., St. Louis, MO, USA, #T6074) membranes were washed with T-TBS (4 times/5 min) and incubated with secondary antibody (Anti-Rabbit or Anti-Mouse at a concentration of 1:1000) for 2 h at room temperature (22 °C ± 2). The proteins were detected by chemiluminescence with the aid of the GE-LAS 4000 photodocumenter (GE Healthcare Life Sciences, Chicago, FL, USA). The data were quantified as the ratio between the optical density of the protein of interest and α-Tubulin on the same blot. Four animals per group were used. The results are expressed as a percentage of the control group (Sham + Sal).

### 2.6. Immunofluorescence Studies

The animals were anesthetized and then submitted to perfusion with 0.9% saline solution, followed by 4% PFA in 0.1 M PBS, for brain fixation. Fixed tissues were cut into 35 µm thick coronal slices with a Vibratome (VT1000S; Leica, Nussloch, Germany). For each animal, three transverse cerebral slices of the neocortex and hippocampus were immunostained [48]. Slices were incubated overnight at 4 °C with the antibodies rabbit anti-GFAP (1:500, Sigma-Aldrich Co., St. Louis, MO, USA, #69269) and mouse anti-NeuN (1:500, Merck Millipore Co., Darmstadt, Germany, #MAB377). Following the incubation with the primary antibody, brain sections were incubated at room temperature for 120 min with a secondary antibody conjugated to fluorescent probes (1:500, Thermo Fisher Scientific Co., Waltham, MA, USA). After that, the sections were assembled with fluoromount (Sigma-Aldrich Co., St. Louis, MO, USA) and imaged in an Olympus FV300 confocal microscope equipped with 488 and 555 nm lasers. The Fiji software was used to measure the fluorescence intensity of GFAP and NeuN (400× magnification); the Region of Interest, ROI, was 223 × 310 µm^2^ for the hippocampus and 265 × 260 µm^2^ for the cortex. A total of 4–6 animals per group were used.

### 2.7. Behavioral Assessment

Behavioral testing in the Open Field, the Elevated Plus Maze, and the Morris Water Maze tasks were performed from PND 45. A total of forty-eight rats were evaluated in the behavioral assessment, according to the experimental groups: Sham + Sal (*n* = 12), HI + Sal (*n* = 10), Sham + EPT (*n* = 14), and HI + EPT (*n* = 12). The rats were filmed during the tests and data were analyzed using Any-Maze software.

#### 2.7.1. Open Field Test

The exploratory activity of the animals was evaluated using the open field arena, a square box with the dimension of 50 × 50 × 39 cm^3^ divided into 12 equal quadrants. The animals were placed in the left posterior quadrant, and the time spent in the peripheral/central zone and the numbers of crossings between the quadrants were recorded for 300 s [49].

#### 2.7.2. Elevated plus Maze

This test consists of the assessment of anxious-like behavior and the exploration of the animal. It was performed in a cross-shaped apparatus suspended 500 mm from the floor, with 4 arms that had a length of 400 mm and a diameter of 200 mm, with two open arms and two closed ones (with a height of 300 mm on the walls). The test was performed for 5 min for each animal, conducted by two examiners, and the variables recorded were: time spent in closed and open arms, and also the number of vertical movements such as rearings and head dippings. The animal’s natural behavior was to remain in the closed arms, with open arms being aversive and less explored [50].

#### 2.7.3. Morris Water Maze

The animals’ spatial reference memory was assessed using the Morris Water Maze. This test was carried out in a circular tank 200 cm in diameter, filled with water (temperature ± 23 °C), with a transparent platform submerged 2 cm below the water surface and located in a room with visual clues on the surrounding walls. The platform position remained in the same place throughout the training period and was run for 5 consecutive days. After 24 h of the learning phase, a probe trial was carried out, consisting of a 60 s session without the platform to evaluate the long-term memory. The following variables were analyzed: latencies in the learning phase and latency to find the platform on the probe trial [50].

### 2.8. Brain Volumetric Analysis

After behavioral testing, the animals were anesthetized with Isoflurane and submitted to transcardiac perfusion with saline solution (0.9%), followed by a solution of 4% paraformaldehyde (PFA). The brains were removed and kept in the same paraformaldehyde solution for 24 h. Then, the brains were cryoprotected with a graduation of 15% and 30% sucrose diluted in a phosphate buffer at 4 °C, and later frozen in isopentane cooled in liquid nitrogen (−30 °C) and stored at −80 °C. Using cryostat equipment (CM1850, Leica), 30 μm coronal sections were made with an interval of 300 μm; the slides containing the slices were stained with Hematoxylin and Eosin. A volumetric analysis of the hemisphere, hippocampus, cerebral cortex, and striatum was performed using NIH-ImageJ software. Cavalieri method [51] was used to estimate the relative volumes of the ipsilateral and contralateral sides to the carotid occlusion following the equation: Σ(area of slices) x (inter-slice interval). The index of tissue loss was measured by the ratio of ipsilateral to contralateral areas calculated by the equation: ipsilateral volume (mm^3^)/contralateral volume (mm^3^) [50]. Eight animals per group were used for lesion volume analysis.

### 2.9. Statistics

Statistical analysis was conducted using the Generalized Linear Model performed by the SPSS-21 software. Injury (sham or HI) and treatment (Saline or PTE) were considered as fixed factors, and data normality was confirmed by Kolmogorov–Smirnov test. Data obtained from behavioral tests (Open Field, Elevated Plus Maze, and Morris Water Maze test probe) as well as biochemical and histological analyses were evaluated using a one-way ANOVA, followed by Tukey’s multiple comparisons test. Repeated measures ANOVA was used to assess the results of the acquisition phase of the Morris Water Maze. Once a main effect or an interaction between factors was found, the Tukey post hoc test was used to compare differences between the groups. Significance was accepted whenever *p* < 0.05. Data are expressed as means ± standard error. The significant differences considered relevant and discussed were: differences between HI + Sal animals and the control group (Sham + Sal); differences between HI + PTE animals and their control group (Sham + PTE), and differences between HI + Sal and HI + PTE animals. The number of animals estimated for each technique was based on previous studies with a similar methodology.

## 3. Results

### 3.1. Chemical Composition

The total phenolic content of the aqueous extract of *Plinia trunciflora* employed here is 783.68 ± 3.40 mg GAE/100 g (dry weight), while the anthocyanins content is 302.81 ± 6.95 mg cyanidin-3-O-glycoside (C3G)/100 g (dry weight).

### 3.2. PTE Prevents HI-Induced Disruption of Redox Homeostasis and an Increase in Pro-Inflammatory Interleukin-1β

**Figure 3:**
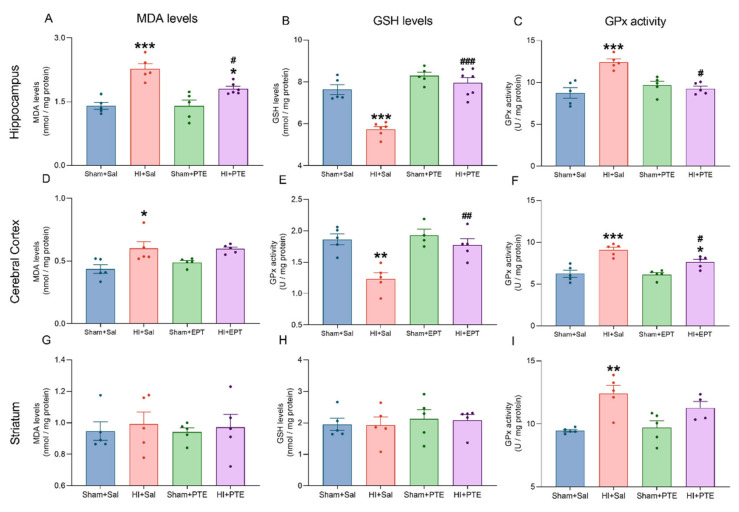
HI-induced oxidative stress biomarker responses. Data are expressed as mean ± SEM. Malondialdehyde (MDA) levels, reduced glutathione (GSH) levels, and glutathione peroxidase (GPx) activity were essayed in the Hippocampus ((**A**–**C**), respectively), in the Cerebral Cortex (**D**–**F**), and in the Striatum (**G**–**I**). * Injury effect (HI vs. control). # Treatment effect (Sal vs. PTE). Sham + Sal (*n* = 4–5); HI + Sal (*n* = 4–6); Sham + PTE (*n* = 4–7); HI + PTE (*n* = 4–7). Significance was accepted whenever *p* < 0.05 (* or #), *p* < 0.01 (** or ##), or *p* < 0.001(*** or ###) (one-way ANOVA).

Oxidative stress and biochemical markers were assessed in the ipsilateral hippocampus, cerebral cortex, and striatum 72 h following neonatal HI; they are presented in Figure 3. MDA levels in the hippocampus revealed an increase in lipoperoxidation in the HI + Sal group as compared to controls (F_(3.17)_ = 15.81 *p* < 0.001), and this effect was prevented in the HI + PTE group (*p* < 0.05); on the other hand, in the cerebral cortex, high lipoperoxidation levels were not prevented by PTE (F_(3.16)_ = 5.733, *p* < 0.05). However, no significant differences were observed between MAD levels among groups when striatum was analyzed (F_(3.16)_ = 0.1257, *p* > 0.05). These findings indicate that HI causes lipid damage in the hippocampus and cerebral cortex, and that PTE prevented lipoperoxidation in these structures.

As regards antioxidant defenses, HI significantly reduced GSH levels in the hippocampus (F_(3.19)_ = 30.62, *p* < 0.001) and cerebral cortex (F_(3.15)_ = 11.27, *p* < 0.001). It was also seen that PTE prevented HI-induced GSH decrease in both brain structures. Again, there were no differences in GSH levels in the striatum (F_(3.16)_ = 0.1748, *p* > 0.05). The activity of antioxidant enzymes was also evaluated. HI increased GPx activity in the hippocampus (F_(3.16)_ = 12.79, *p* < 0.001), cerebral cortex (F_(3.16)_ = 17.09, *p* < 0.001), and striatum (F_(3.15)_ = 8.222, *p* < 0.001). As predicted, HI + PTE rats exhibited enzyme activity comparable to that of controls in the hippocampus and in the cerebral cortex. There were no differences between groups of SOD activity evaluated in the hippocampus (F_(3.20)_ = 0.9650, *p* > 0.05), cerebral cortex (F_(3.15)_ = 2.115, *p* > 0.05), and striatum (F_(3.15)_ = 0.4790, *p* > 0.05). These results demonstrate that PTE was able to reestablish the levels of antioxidant defenses affected by HI.

**Figure 4:**
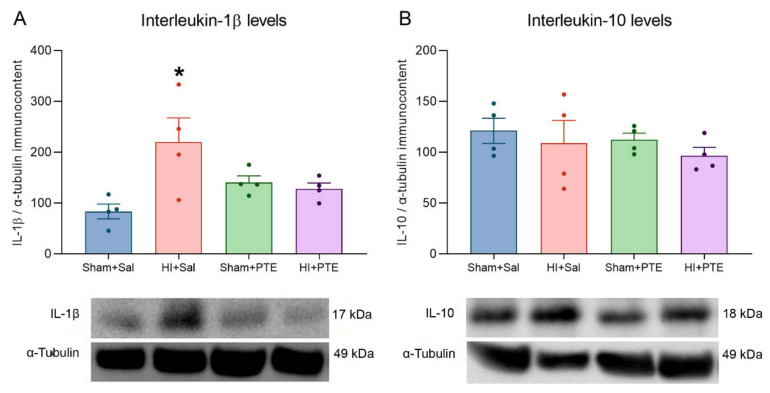
Interleukin immunocontent level assessment in the right hippocampus 72 h after neonatal HI and representative protein blots. Data are expressed as mean ± SEM. Interleukin-1β (IL-1β) levels (**A**). Interleukins-10 (IL-10) (**B**). Results are plotted relative to the same sample used in all membranes (expression level considered as 100%) and normalized by α-Tubulin (mean ± SEM). * Injury effect (HI vs. control). *n* = 4 animals per group. Significance was accepted at *p* < 0.05 (*) (one-way ANOVA).

The immunocontent of interleukins evaluated in the right hippocampus (ipsilateral to the carotid occlusion) are presented in Figure 4. HI increased interleukin-1β levels as compared to the controls (F_(3.13)_ = 3.800, *p* > 0.05); this effect was prevented in HI rats pre-treated with PTE. Interleukin-10 levels did not vary between experimental groups (F_(3.14)_ = 0.3567, *p* > 0.05).

### 3.3. PTE Prevents HI-Induced Neuronal Loss and Greater GFAP Immunoreactivity

**Figure 5:**
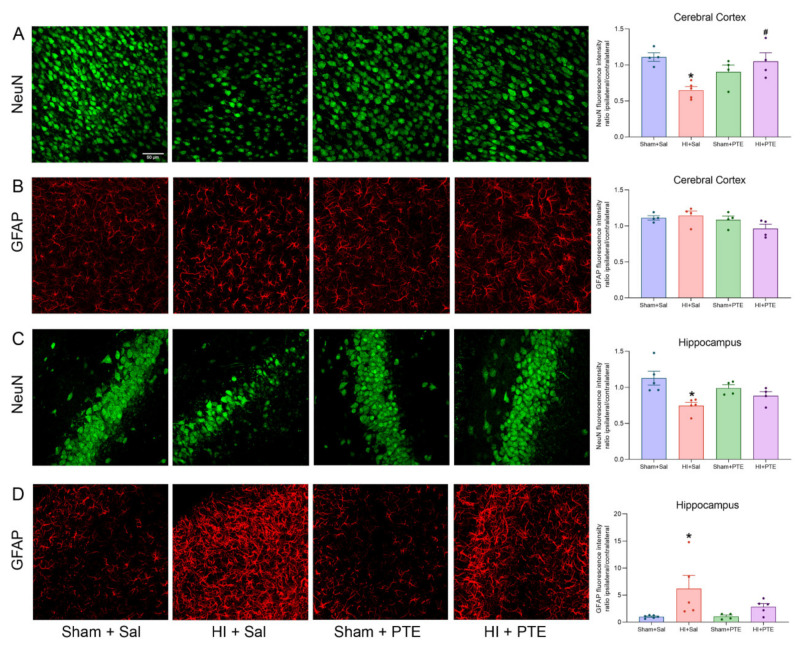
Representative immunofluorescence images showing NeuN (**A**) and GFAP (**B**) staining in the cerebral cortex; NeuN (**C**) and GFAP (**D**) staining in the hippocampus 72 h following neonatal HI. Data are expressed as mean ± SEM. Quantification of the fluorescence intensity of NeuN and GFAP was performed at 400× magnification by using the mean of five randomly selected fields from each brain. * Injury effect (HI vs. control). # Treatment effect (Sal vs. PTE). Sham + Sal (*n* = 4–6); HI + Sal (*n* = 4–5); Sham + PTE (*n* = 4); HI + PTE (*n* = 4–5). Significance was accepted at *p* < 0.05 (* or #) (one-way ANOVA).

The extension of neuronal injury and glial reactivity caused by HI and the PTE preventive effects are depicted in Figure 5. The fluorescence intensity ratio between the ipsilateral/contralateral hippocampus following HI demonstrates a reduction of NeuN levels in HI + Sal animals (F_(3.14)_ = 6.095, *p* < 0.05), indicating neuronal loss. HI-induced NeuN reduction was also observed in the cerebral cortex (F_(3.13)_ = 6.700, *p* < 0.05); conversely, the effect was abolished in HI rats treated with PTE (*p* < 0.05). HI also caused astrocyte reactivity in hippocampus, as assessed by the marked increase in the fluorescence intensity of GFAP (F_(3.16)_ = 3.744, *p* < 0.05). As predicted, such increase was not observed in HI animals receiving PTE (*p* > 0.05). In contrast, no difference was observed in GFAP intensity in the cerebral cortex (F_(3.12)_ = 2.214, *p* > 0.05).

### 3.4. PTE Partially Prevents HI-Induced Behavioral Impairments and Brain Tissue Damage Assessed at Adult Age

Exploration and locomotion of the animals in the Open Field and the Elevated Plus Maze tasks are presented in Table 1. No significant differences were observed in the time spent in the central (F_(3.44)_ = 1.220, *p* > 0.05) or peripheral (F_(3.44)_ = 1.092, *p* > 0.05) areas; however, both HI groups had a higher number of crosses between the quadrants as compared to control animals (F_(3.44)_ = 10.33, *p* < 0.001) in the Open Field task. One-way ANOVA of the Elevated Plus Maze test showed that HI + Sal animals remained longer in the open arm (F_(3.44)_ = 5.689, *p* < 0.05) and had an increased ratio for the open/closed arm (F_(3, 44)_ = 5.360, *p* < 0.05). HI animals receiving PTE displayed similar behaviors to those of controls on both measures (*p* > 0.05). Additionally, analysis showed that HI + Sal animals presented more head dipping (F_(3.44)_ = 2.810, *p* < 0.05), and that both HI groups presented a greater number of rearing responses (F_(3.44)_ = 5.247, *p* < 0.05).

**Figure 6:**
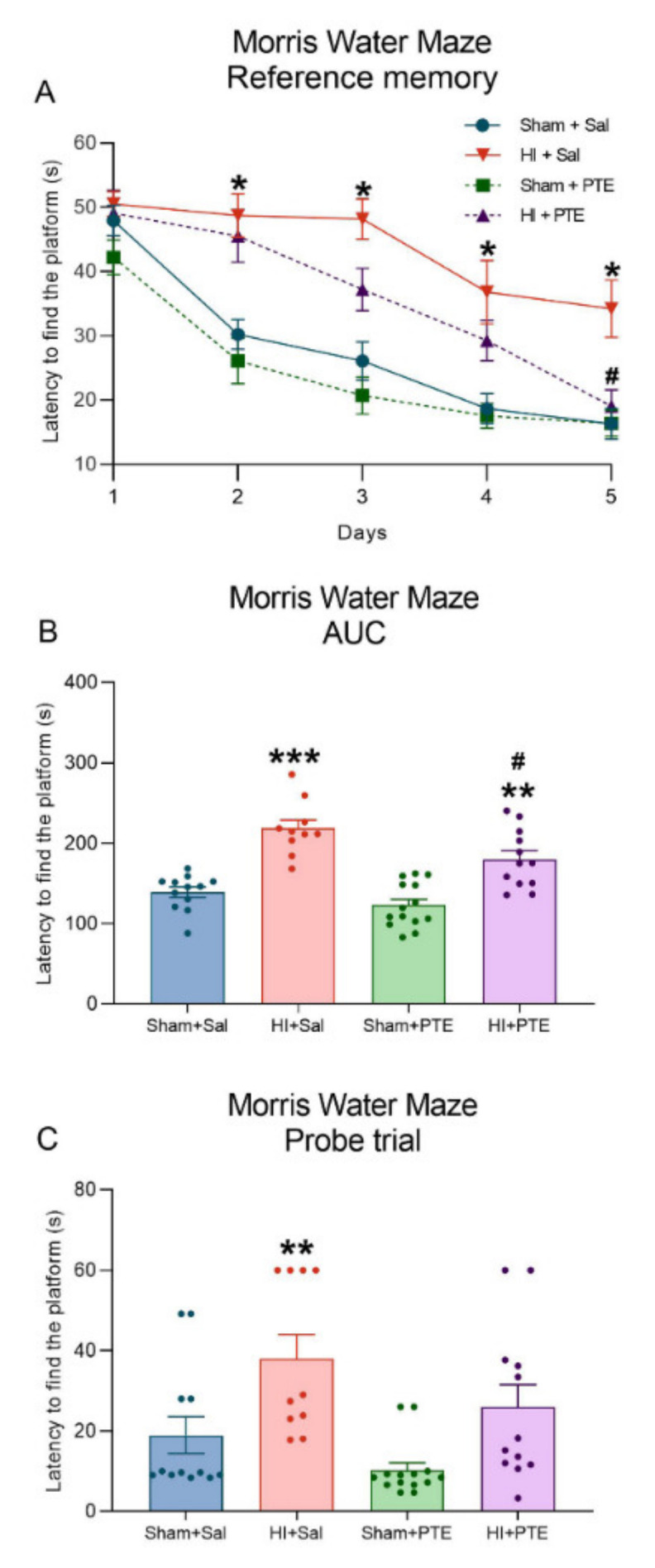
Reference spatial memory performance in the Morris Water Maze assessed at adult age. Data are expressed as mean ± SEM. Average latencies to reach platform during five consecutive days of training (**A**). Area under the curve of latency to find the platform during training (**B**). Latency to cross the platform location in the probe trial (**C**). * Injury effect (HI vs. control). # Treatment effect (Sal vs. PTE). Sham + Sal (*n* = 12); HI + Sal (*n* = 10); Sham + PTE (*n* = 14); HI + PTE (*n* = 12). Significance was accepted whenever *p* < 0.05 (* or #), *p* < 0.01 (** or ##), or *p* < 0.001 (*** or ###) (one-way ANOVA).

Spatial reference memory data are plotted in Figure 6. Repeated measures ANOVA of the Morris Water Maze revealed that HI + Sal animals presented cognitive deficits related to the acquisition of reference memory (F_(3.44)_ = 23.068, *p* < 0.001). One-way ANOVA showed that HI + Sal animals had increased latencies to find the platform on trials 2 (F_(3.44)_ = 5.091, *p* < 0.01), 3 (F_(3.44)_ = 4.578, *p* < 0.001), 4 (F_(3.44)_ = 4.519, *p* < 0.001), and 5 (F_(3.44)_ = 4.166, *p* < 0.001) as compared to controls. Interestingly, HI animals treated with PTE had a learning curve with shorter trial 5 latency as compared to that of HI + Sal animals (F_(3.44)_ = 4.168, *p* < 0.001). Analysis of the area under the curve showed a clear HI effect on memory acquisition, an effect partially prevented by PTE administration (F_(3.44)_ = 23.07, *p* < 0.001). The memory retention index analysis on the probe trial showed that HI + Sal animals had a higher latency to cross the platform area (F_(3.44)_ = 6.463, *p* < 0.001), whereas the HI + PTE group did not differ from control animals, indicating that HI injury hinders spatial learning and memory processes, and that PTE is able to preserve the ability of injured rats to acquire and store new memories.

**Figure 7:**
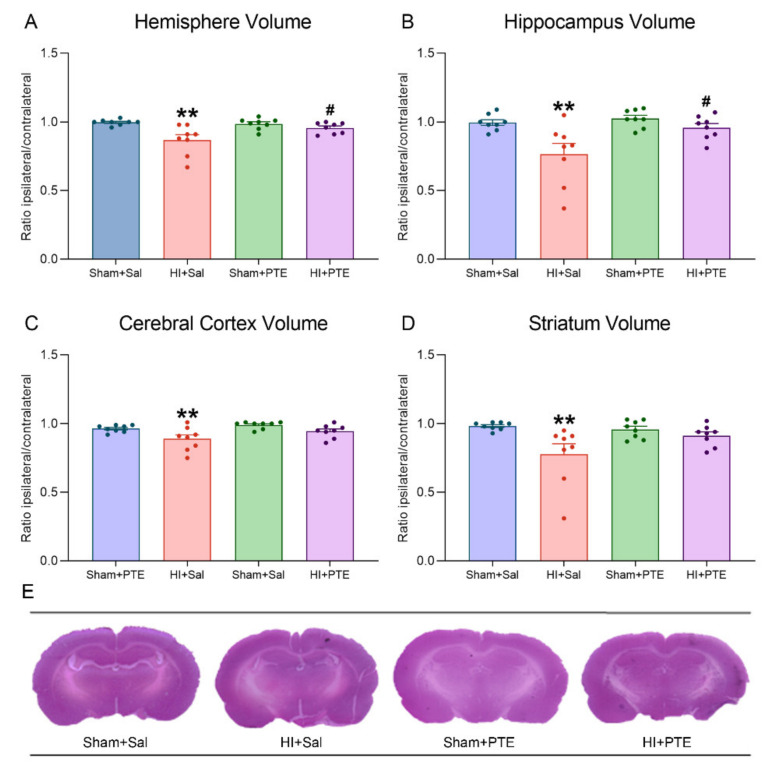
Ipsilateral/contralateral volume ratio of the hemisphere (**A**), the hippocampus (**B**), the cerebral cortex (**C**), and the striatum (**D**). Representative hematoxylin and eosin-stained coronal brain sections (Bregma −3.3 mm) of adult rats (**E**). Data were calculated by dividing the volume of selected brain structure ipsilateral to the carotid occlusion (right side) by the contralateral volume. Data are expressed as mean ± SEM. * Injury effect (HI vs. control). # Treatment effect (Sal vs. PTE). *n* = 8 animals per group. Significance was accepted whenever *p* < 0.05 (* or #) or *p* < 0.01 (** or ##) (one-way ANOVA).

Neonatal HI impacts on brain tissue assessed in adult rats following the behavioral experiments are pictured in Figure 7. The volumetric ratio of brain structures (ipsilateral/contralateral to HI injury) was shown to be reduced in the hemisphere (F_(3.28)_ = 7.072, *p* < 0.01), in the hippocampus (F_(3.28)_ = 6.877, *p* < 0.01), in the cerebral cortex (F_(3.28)_ = 5.417, *p* < 0.01), and in the striatum (F_(3.28)_ = 4.650, *p* < 0.01). Interestingly, HI rats receiving PTE showed significant differences as compared to HI + Sal in the hemisphere (*p* < 0.05) and the hippocampus (*p* < 0.05) and did not differ from controls in the cerebral cortex and the striatum (both *p* > 0.05), evidencing the neuroprotective properties of the PTE.

## 4. Discussion

The present study provides evidence of the beneficial effects of PTE administration after neonatal HI in Wistar rats. Confirming the working hypothesis, HI animals that received i.p. 10 mg/kg of PTE did not exhibit HI-induced redox imbalance, pro-inflammatory interleukin increase, reactive astrogliosis, and neuronal death 72 h after the injury event. In addition, PTE administration was able to partially prevent spatial reference memory deficit, attenuate anxious-like behavior, and preserve brain tissue in HI rats assessed at adult age. Here, we were able to demonstrate PTE-mediated neuroprotective effects against neonatal HI injury for the first time.

*Plinia trunciflora* extract is composed of anthocyanins, phenolic compounds belonging to the class of flavonoids [31]; the presence of C3G anthocyanins was confirmed in the extract used in this work. The pharmacokinetic characteristics of anthocyanins are quite well-understood, their absorption occurs through glycosides in both rodents and humans, and once absorbed, anthocyanins can cross the blood–brain barrier and reach the nervous parenchyma [52,53]. Evidence suggests that dietary intake of anthocyanins-rich fruits are associated with a decrease in brain damage caused by Alzheimer’s disease [54], Parkinson’s disease [55], and cerebral ischemia during adulthood [56]. The possible neuroprotective mechanism mediated by anthocyanins and C3G is the suppression of neuroinflammation and oxidative stress [57].

Results presented here indicate that neonatal HI injury increased lipoperoxidation levels in the hippocampus and the cerebral cortex. Phospholipid peroxidation of membranes leads to cell damage, which is one of the most important effects of oxidative damage [8]. HI animals are more susceptible to lipid damage due to the increased production of reactive oxygen species resulting from mitochondrial failure [9], which could also underlie vulnerability to age-dependent injury [58]. Furthermore, it was observed that PTE prevented lipoperoxidation in the cerebral cortex and attenuated this effect in the hippocampus, well in accordance with previous reports on a PTE dose-dependent effect on lipid damage, in both in vivo and in vitro studies [31,38].

The evaluation of oxidative parameters did not show alterations in the activity of the SOD enzyme in the hippocampus, the cortex, and the striatum ipsilateral to the lesion. On the other hand, we observed an increase in GPx enzyme activity and a decrease in the concentration of the antioxidant molecule GSH in the HI animals; PTE treatment was able to prevent these effects in the hippocampus, and partially in the cerebral cortex and striatum. Along this line, it was reported that C3G demonstrates neuroprotective effects related to the suppression of oxidative stress, such as the reduction of lipoperoxidation and increased levels of the nonprotein thiol group, heme oxygenase-1 (HO-1), and gamma-glutamyl cysteine synthase (γ-GCS) in the cerebral tissues of rats with cerebral ischemia [59].

Previous reports have demonstrated that neonatal HI displays a regional brain vulnerability associated with the stages of brain development and injury severity [58,60]. Hippocampal pyramidal cells have shown a higher susceptibility to HI insult when compared to other brain structures such as the cerebral cortex or the striatum [61,62], in agreement with the results presented here. Moreover, this structure is the most studied regarding biochemical, cellular, and functional damage following HI insult; hence, the focus on the hippocampus. Despite the striatum and the cerebral cortex being affected, the hypoxia exposure used (8% O_2_ for 60 min) can be considered as mild to moderate when HI was induced at PND 7, which spared these brain structures, minimizing evidence of damage. Studies that used natural polyphenol compounds showed a reduction in brain damage after neonatal HI, indicating a higher neuroprotection in the hippocampus [26] and a protective effect, possibly as a consequence of the attenuation of cell death induced by oxidative damage [63].

Neuroinflammation is a key feature of brain damage after HI insult, which occurs through the interaction with several immune pathways such as interleukin responses [64]. Furthermore, basal levels of several cytokines show age-related differences. For example, the newborn hippocampus exhibits a six-fold higher interleukin-1β (IL-1β) level when compared to adult brains, impacting the innate immune response to injury [58]. Damaged neurons produce several cytokines, including interleukins (IL-1, IL-6, IL-8) and TNF-α, which activate the inflammatory response and trigger biochemical processes that lead to secondary energy failure and the death of neuronal cells [65,66]. Anthocyanins such as C3G also have the great anti-inflammatory potential [67,68]. The study of these mechanisms demonstrates that anthocyanins can inhibit neuroinflammation through the inactivation of JNK activity and the reduction of NF-Κb activity [69]. In this study, it is possible to observe that HI animals receiving PTE have pro-inflammatory IL-1β levels similar to those of the control animals. However, no changes were observed in IL-10 levels, suggesting that the PTE does not modulate this anti-inflammatory interleukin. Although the results suggest the anti-inflammatory action of PTE, considering IL-1β levels, the results should be interpreted with caution since microglia markers following HI insult were not evaluated in order to confirm if PTE could provide some degree of neuroprotection mediated by microglial attenuation.

Antioxidant and anti-neuroinflammatory effects mediated by C3G anthocyanins, such as those present in PTE, contributed to neuronal survival in cerebral ischemic [56] and Alzheimer’s disease rat models [54]. Furthermore, Williams (2008) [70] demonstrated that the mechanism of this neuroprotective effect may include the activation of protein kinase B (Akt), extracellular signal-regulated kinase 1/2 (ERK1/2), the cAMP-response element binding protein (CREB), and the brain-derived neurotrophic factor (BDNF). In spite of the neuroprotection described after neonatal HI, the PTE is not fully characterized and the main compound or compounds, as well as their synergistic interaction that could be mediating the beneficial effect, are not yet clear.

This study demonstrated a neuronal loss in the hippocampus and the cerebral cortex, and also astrogliosis in the hippocampus ipsilateral to carotid occlusion 72 h following HI. It is well-understood that HI injury leads to neuronal death and astrocytic reactivity through the activation of apoptotic pathways [64]. In addition, it is shown that HI animals receiving PTE maintain many neurons, which could indicate probable neuroprotection caused by the extract. Based on this, the effects of PTE on CNS functionality was investigated in adult rats following HI through behavioral tests.

HI caused hyperlocomotion and anxiety-like behavior since there were more crossings in the Open Field test and an increased ratio of the open/closed arm in the Elevated Plus Maze task. Supporting these findings, Sanches et al. (2013) [50] demonstrated anxiety-like behavior in HI animals on PND3 when the assessment in the Elevated Plus Maze was performed on PND60, and Arteni et al. (2010) [51] observed that male rats submitted to HI on PND7, showing increased levels of anxiety in adulthood (PND90). These authors suggest that HI injury may have caused damage to the catecholaminergic system, leading to the hyperactivity observed in the injured animals [71]. The lack of inhibitory potentials, especially in the frontal cortex, could explain the impulsive behavior with the high open/closed ratio, exceeding the natural behavior of rats to spend time in the closed arm [50,51]. Otherwise, PTE-treated animals showed a similar open/closed ratio to control animals, indicating the possible modulation of anxiety-like behavior. In this context, Sacchet et al. (2015) [38] demonstrated that PTE promoted anxiolytic effects in an animal model of stressful situations.

Spatial memory deficits caused by HI are well-described in the literature [49,72,73]. In this study, it was observed that HI animals receiving PTE had a lower latency to find the platform than HI animals during training. However, considering the area under the curve (AUC), it was noted that PTE treatment partially decreased spatial memory impairment caused by HI. Spatial memory impairments are attributable to tissue damage caused by the HI event, namely lesions to the hippocampus, the striatum, and the cerebral cortex. As predicted, PTE administration preserved neural tissue in all the three structures studied; all are known to have participated in spatial memory processing.

## 5. Conclusions

The present study shows that treatment with the *Plinia trunciflora* fruit extract results in brain tissue preservation and the prevention of HI-induced spatial memory impairment, possibly due to the attenuation of oxidative stress damage, the reduction of inflammatory response, and the decrease of neuronal death and astrogliosis reactivity in the CA1 subfield of the hippocampus. Since plant extracts have a large chemical variety, we cannot elect the specific compounds responsible for the therapeutic effects observed in this study. Among the compounds present in PTE, anthocyanins have a proven neuroprotective action. This makes it tempting to suggest that our results may have, at least in part, a contribution from this compound. Additional studies should clarify and confirm the therapeutic potential of the *Plinia trunciflora* extract against the deleterious effects of neonatal hypoxia–ischemia.

## Figures and Tables

**Table 1:** Open Field and Elevated Plus Maze performance. Data are expressed as means ± SE. * Injury effect (HI vs. control). Sham + Sal (*n* = 12); HI + Sal (*n* = 10); Sham + PTE (*n* = 14); HI + PTE (*n* = 12). Significance was accepted whenever *p* < 0.05 (* or #), *p* < 0.01 (** or ##), or *p* < 0.001 (*** or ###) (one-way ANOVA).

	Sham + Sal	HI + Sal	Sham + PTE	HI + PTE
**Open Field test**				
Time spent in central zone	12.76 ± 1.99	15.59 ± 2.76	16.81 ± 2.49	11.46 ± 1.90
Time spent in peripheral zone	287.2 ± 1.99	284.4 ± 2.76	283.2 ± 2.49	288.3 ± 2.06
Crossing	114.3 ± 6.74	175.2 ± 12.67 ***	145.4 ± 4.12	153.6 ± 7.07 *
**Plus Maze**				
Time spent in open arms	47.33 ± 7.00	93.4 ± 13.06 *	53.5 ± 7.19	64.17 ± 5.12
Time spent in closed arms	189.0 ± 9.74	155.1 ± 19.1	192.7 ± 9.77	154.8 ± 11.71
Ratio time in open/closed arms	0.25 ± 0.04	0.75 ± 0.19 *	0.31 ± 0.05	0.45 ± 0.05
Rearings	6.41 ± 0.58	10.6 ± 1.25 *	8.42 ± 1.05	11.83 ± 1.16 *
Head dipping	2.66 ± 0.46	7.6 ± 2.17 *	3.85 ± 0.62	5.41 ± 1.39
* Injury effect (HI vs. control)				

## Data Availability

The datasets generated and/or analyzed during the current study are available from the corresponding author on reasonable request.
